# Eyewitness Memory in Face-to-Face and Immersive Avatar-to-Avatar Contexts

**DOI:** 10.3389/fpsyg.2018.00507

**Published:** 2018-04-17

**Authors:** Donna A. Taylor, Coral J. Dando

**Affiliations:** Department of Psychology, University of Westminster, London, United Kingdom

**Keywords:** avatar-to-avatar communication, eyewitness testimony, episodic memory, virtual environment, cognition in context

## Abstract

Technological advances offer possibilities for innovation in the way eyewitness testimony is elicited. Typically, this occurs face-to-face. We investigated whether a virtual environment, where interviewer and eyewitness communicate as avatars, might confer advantages by attenuating the social and situational demands of a face-to-face interview, releasing more cognitive resources for invoking episodic retrieval mode. In conditions of intentional encoding, eyewitnesses were interviewed 48 h later, either face-to-face or in a virtual environment (*N* = 38). Participants in the virtual environment significantly outperformed those interviewed face-to-face on all episodic performance measures – improved correct reporting reduced errors, and increased accuracy. Participants reported finding it easier to admit not remembering event information to the avatar, and finding the avatar easier to talk to. These novel findings, and our pattern of retrieval results indicates the potential of avatar-to-avatar communication in virtual environments, and provide impetus for further research investigating eyewitness cognition in contemporary retrieval contexts.

## Introduction

Technological innovations offer exciting possibilities for changes in practice within the criminal justice system. One application area is the way in which witness^1^ information is elicited. Witnesses provide information about personal experiences within a specific context (Tulving and Thomson, 1973), which requires conscious recollection and is contingent on a type of mental set referred to as episodic ‘retrieval mode’ (Burgess et al., 2002; Tulving, 2002; Roediger et al., 2014). Witness information is typically collected during a face-to-face interview (e.g., Ministry of Justice (MOJ), 2011; Cooper and Norton, 2017), and so for witnesses, episodic retrieval mode occurs within a social environment.

Recalling and recounting an experienced event is cognitively demanding. Witnesses complete a series of complex cognitive operations to activate episodic retrieval (e.g., Tulving and Thomson, 1973; Schacter et al., 1997), which requires effort and concentration, because unlike semantic memory, for example, episodic retrieval is not automatic. In addition to the cognition specific demands, interviews are social interactions, so witnesses also have to manage the social elements of the retrieval environment, including social signals arising during an interview that may implicitly influence their cognition (e.g., Fiske and Taylor, 2013). It is the retrieval environment, and its impact on cognition that is the focus of the research reported here. Using a mock witness paradigm, we investigated whether episodic retrieval in an immersive virtual environment confers advantages by attenuating the social situational demands of a face-to-face retrieval (Wells, 1978; Perfect et al., 2008) thereby reducing task related perceptual load (e.g., Murphy and Greene, 2016), releasing more cognitive resources for invoking episodic retrieval.

### Situational Task Demands

Being questioned about a crime event by one or two police officers, at a police station or elsewhere, can be intimidating, and stressful (Ministry of Justice (MOJ), 2011). The imbalance of power and control (perceived or actual), and witness anxiety to perform well can render them passive and cautious (e.g., Haworth, 2006; Fisher, 2010), responding *only* to questions asked, not always volunteering additional information, and holding back partially remembered information to avoid appearing foolish. Conversely, anxiety to perform well can result in the reporting of erroneous information. Even when witnesses are unsure, have not encoded the requested information, or experience a retrieval failure, the demand characteristics of a face-to-face interview can result in witnesses guessing, acquiescing, and/or reporting script consistent, but nonetheless incorrect information (e.g., Blank and Launay, 2014; Harlow and Yonelinas, 2016; Fisher and Schreiber, 2017).

The presence of ‘warnings’ during an interview, such as not to guess or not to answer questions if unsure, can reduce some of the errors described (Fisher, 2010; Geiselman and Fisher, 2014). Likewise, rapport building can improve witness performance, reducing misinformation and inaccuracies, particularly in free recall accounts (see Vallano and Compo, 2011; Kieckhaefer et al., 2014). The physical presence of others can also interfere with accuracy of recall (Bond and Titus, 1983). Correct responses to complex questions decrease as the number of persons present increases (Wagstaff, 2008). Conversely, minimizing physical contact can improve the amount of information reported (Powell et al., 2002), the suggestion being that when external interference is reduced witnesses can fully concentrate on activating episodic retrieval mode (Murphy and Greene, 2016).

Eye contact has been found to disrupt cognitive performance whereby maintaining eye contact during a cognitive task can mitigate performance vs. no eye contact and eyes closed task conditions (e.g., Markson and Paterson, 2009; Buchanan et al., 2014), although environmental distraction may not always impair performance (e.g., Rae and Perfect, 2014). Witnesses interviewed remotely have reported a reduction in the perceived social pressure to ‘perform,’ and have performed equally to those interviewed face-to-face, indicating that physical co-presence may not be necessary (Nash et al., 2014). Similarly, children interviewed remotely using Skype Video-Mediated Communication (VMC) provided equally as informative and accurate accounts as those interviewed face-to-face (Hamilton et al., 2017), and in some cases VMC reduced errors and susceptibility to leading questions (Doherty-Sneddon and McAuley, 2000).

### Virtual Environments

Virtual environments (VEs) are computer simulations that represent activities at a high degree of realism (see Witmer and Singer, 1998). Virtual environments are easily created and managed using portable computer technology, which can render visual, auditory, and haptic information to users within milliseconds. VEs can bring about realistic behavior and responses because the environment ‘feels’ real, often bringing about physiological responses to environmental challenges and changes (see Slater, 2009; Gonzalez-Franco and Lanier, 2017). Thus, VEs offer potential as interviewing spaces. Research from psychological, military and medical domains indicates potential advantages of VEs for gathering witness information. For example, in simulated child sex abuse interviews with computer generated avatars, feedback on interviewing performance quickly improved interviewing techniques, and enhanced interview outcomes (Pompedda et al., 2015). Anonymity can encourage disclosure (e.g., Joinson, 2001; Suler, 2004) and reduce performance anxiety, which can mitigate risks such as rejection by listeners, reduction of personal integrity, loss of control and embarrassment (e.g., Rubin, 1975; Omarzu, 2000). VEs can also encourage more flexible, innovative and efficient cognition (Isen, 2001; Kalyuga and Liu, 2015), and facilitate systematic and careful processing of task information (Carnevale and Isen, 1986; Spector, 2014).

Virtual environments allow people to communicate as *avatars*, which are digital models or visual projections that represent a synthetic reality (Bailenson et al., 2001; Ahn et al., 2013). Avatars allow changes in physical attributes such as race, gender, age, and physical disabilities, so stereotypical behaviors arising from biased first impressions that negatively impact performance in face-to-face interviews can be avoided (Dubrovsky et al., 1991; Matheson, 1991). Importantly, people appear to interact with avatars in a manner that indicates they are received similarly to real people (Kilteni et al., 2015; Gonzalez-Franco et al., 2016). Indeed, avatar-to-avatar (AtoA) communication has been found to confer cognitive benefits, resulting in improved customer engagement (Verhagen et al., 2015), higher levels of cooperation, and reductions in the amount of communication required to achieve efficient outcomes (Greiner et al., 2014). AtoA communication can improve learning outcomes despite ratings of low social presence (McKerlich et al., 2011), and higher levels of confidence between avatar learners and teachers in VEs can bring about improved productivity (e.g., Salmon et al., 2010).

The potential of VEs is extended by the introduction of avatars in place of human roles. Avatar-based interview simulators allow free-flowing conversation, and can create realistic interactive experiences (Kuykendall, 2010; Pompedda et al., 2015). Junior doctors can practice surgical and diagnostic techniques before interacting with real-life patients (Teteris et al., 2012), and head-mounted displays create immersive experiences for military situation awareness and decision-making training (Cruz-Neira et al., 2011). Accordingly, immersion may offer opportunities to reduce the situational task demands of face-to-face witness interviews. Yet, despite an increasing body of research investigating VEs and AtoA communication in other domains, the use of VEs for gathering witness information has yet to be investigated.

### The Present Study

We investigated episodic performance during interviews conducted in an AtoA context, compared to a traditional face-to-face interview. We hypothesized that interviewing participants in a VE where the interviewer and interviewee are represented by avatars may improve episodic recall compared to a face-to-face interview for two reasons. First, the demand characteristics associated with the physical presence of the interviewer, inherent in face-to-face interviews (Fisher, 2010), may be attenuated, which may reduce errors emanating from real or perceived pressure to perform. Second, witnesses interviewed in VEs do not have to attend to the situational dynamics of the interview context, and so are not ‘dual tasking’ (Perfect et al., 2008, 2012), hence, more cognitive resources may be available to facilitate episodic retrieval.

## Materials and Methods

### Participants

Thirty-eight adults from the general population took part in the research (*M*_age_ = 24.92 years, *SD* = 4.76, ranging from 18 to 38 years), 26 females and 12 males, of which 20 participants (14 female; 6 male) were randomly assigned to a face-to-face (FtoF) condition, and 18 (12 female; 6 male) were allocated to an AtoA condition. Participants were recruited using social media (Facebook; Twitter) or by word of mouth. This research was approved by the University of Westminster Psychology Ethics Committee, and run in accordance with the British Psychological Society code of ethical conduct.

### Materials and Equipment

A pre-recorded video of a mock crime event lasting approximately 1 min 45 s was presented via a laptop computer. The film depicted the theft of a car left unattended by the driver with the window open. The perpetrator accessed the car by leaning through the open window. He started the car, and drove it across town, searching through the contents of the car including the owner’s wallet as he drove. He then parked the car in a residential area.

Post-video interviews were structured according to the UK investigative interview model (PEACE) and Achieving Best Evidence advice (Ministry of Justice (MOJ), 2011). Irrespective of retrieval condition, all interviews followed a fixed sequence of phases: (i) greet, (ii) explain, (iii) free recall, (iv) probed recall, and (v) closure [full interview protocols are available from the first author – also see Ministry of Justice (MOJ) (2011) for information on greet, explain and closure phases of the interviews]. The explain phase included four ground rules used in a UK police Tier 1 basic witness interview, which includes the following ground rules: Never Guess; Report everything; Say if you do not remember; and tell me if you do not understand the question. The theoretical and empirical rapport building literature is sparse, and provides limited guidance on what actually constitutes rapport (e.g., Vallano and Schreiber Compo, 2015), hence rapport building in forensic contexts is not well understood, and is variously and loosely described (Vallano and Compo, 2011; Walsh and Bull, 2012; Abbe and Brandon, 2013, 2014). Accordingly, the protocols developed for this research did not include a rapport building phase, as such. Rather, the interviewer interacted and conversed with each participant during both the greet and explain phases prior to moving to the more formal retrieval phases. During the greet phase participants were asked by the interviewer whether they had taken part in research before, which typically initiated a short conversation prior to moving to the explain phase.

Free recall was initiated using an open invitation: ‘tell me everything you can remember about the video you saw a couple of days ago.’ Participants provided their initial account uninterrupted by the interviewer, who waited a further 10 s after the participant had stopped speaking before moving on to the next phase. During this initial account, the interviewer made bullet point notes about each of the topics mentioned by the participant in the order that they were mentioned. These notes were then used to guide the questioning phase so as to ensure witness-compatible questioning. Participants were reminded of the four interview rules, and then questioned about each topic in turn, first with an initial open-ended invitation to ‘tell me everything about ….’, followed by a series of probing questions (using Who; What; Why; When; Where; How), as appropriate to the interviewee’s response.

A post-interview questionnaire was administered (see **Table 2**) concerning perceptions of their performance, and experience of participation. Seven questions were answered by all participants, appropriately worded according to condition (e.g., in the AtoA condition, the term ‘interviewer’ was replaced by ‘avatar’). Three additional questions were included in the feedback for participants in the AtoA condition only, and concerned their experience of being interviewed in a VE.

**FIGURE 1:**
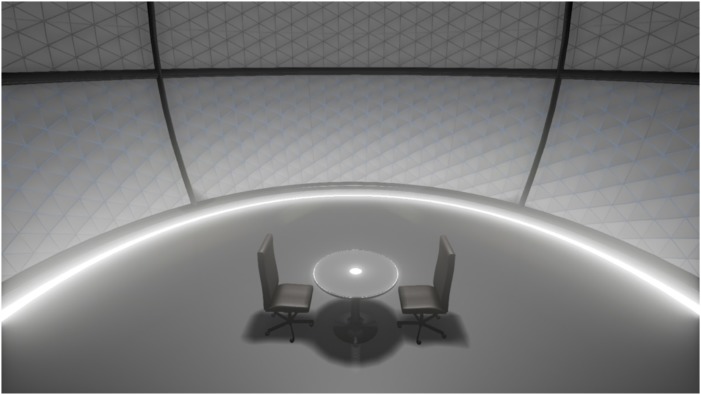
Birdseye view of the AtoA virtual interview environment.

In the AtoA condition, the interviewer and participant were located in different rooms within the same building, and communicated using an Oculus Rift virtual reality headset. The Oculus Rift was designed to create a sense of complete immersion in a three-dimensional world and has 1920 × 1080 high resolution OLED panels, one for each eye, which globally refresh at a rate of 90 Hz. An on-board Inertia Measurement Unit (IMU) positional camera allowed transitional and rotational movement to be tracked. Verbal communication was via a headset with DAC (digital-to-analog converter) to provide a 3D audio effect. The virtual environments were displayed on Intel Core i7-4720HQ, 2.60GHz CPU Windows 8.1, 64-bit NVIDIA GeForce GTX 980M Graphics Card, 16.0 GB of RAM 250 GB SSD. A bespoke, virtual interview environment was designed for this research using Unreal Engine 4. The research environment was purposely sparse, comprising a table with a chair either side – one for the avatar interviewer, the other for the avatar participant (see **Figure 1**). Participants did not experience complete embodiment, but head movements were tracked, and so were experienced by participants.

### Procedure

**FIGURE 2:**
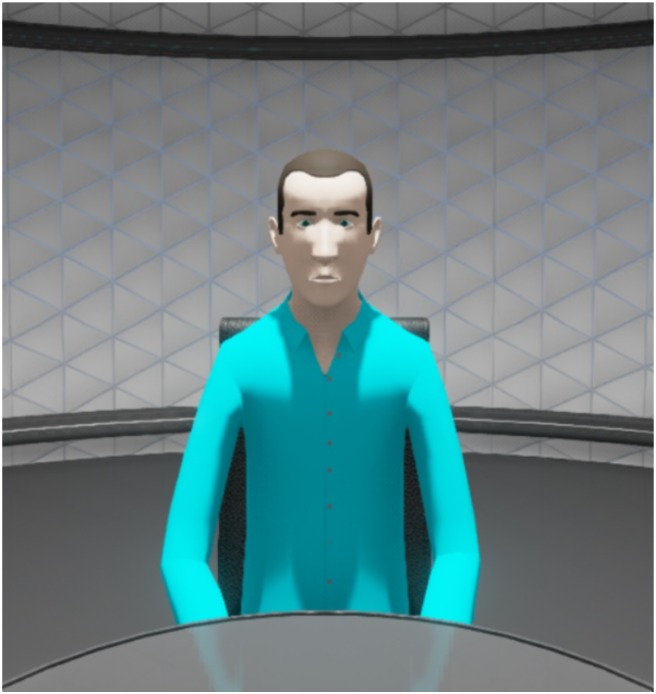
Participant view during the interview.

Participants were recruited to take part in research purportedly testing the Oculus Rift headset (AtoA condition), and so were naïve to the real aims of the project. All participants individually viewed the mock crime stimulus event on a laptop computer, and only after having viewed the film were participants then informed that they would be asked to provide some information about the film 2 days later. Each participant was then randomly allocated to one of two retrieval conditions – FtoF or AtoA. Participants were individually interviewed on University premises 48 h after watching the video. Irrespective of condition, all interviews were conducted by one of two female interviewers (A and B) using the interview protocol described above. In the FtoF condition participants were interviewed by interviewer A in a room with a table and two chairs configured similarly to the VE, with no additional objects (the digital voice recorder was not visible). In the AtoA condition, participants were interviewed by interviewer B with both participant and interviewer communicating as avatars – they did not meet face-to-face until after the interview had been conducted. Using the Oculus Rift headset, interviewer and interviewee were exposed to the VE, and were presented with a basic avatar menu, which allowed them to choose either the male or female avatar. From that point on, interviewer and participant communicated via the Oculus Rift, and viewed each other as avatars throughout the interview (see **Figure 2** for participant view). Interviews in the VE were digitally captured (voice and video) by the Unreal Engine 4 software. Written consent was provided by each participant prior to their participation (before watching the stimulus video). Verbal consent was also gained (and audio recorded) again from each participant, immediately prior to the interviews (48 h later).

### Interview Coding

Interviews were transcribed and coded according to a scoring template technique (e.g., Memon et al., 1996). Each item recalled by participants was scored as correct, erroneous (information relevant to the witnessed episode but described with error, e.g., describing a person’s brown jacket, but stating that it was black instead brown), or confabulated (reporting information that was not present in the film). The position in the interview that the information was recalled was also coded (i.e., whether recalled in the Free Recall or Questioning phases) Items recalled were only scored once (i.e., repetitions were not scored irrespective of interview phase).

Ten interviews (5 AtoA; 5 FtoF) were randomly selected for recoding by an independent coder blind to the aims and hypotheses of the research but familiar with the template method of scoring. Pearson’s correlations for the overall amount of correct, erroneous, and confabulated recall revealed good levels of inter-rater reliability for all measures, *r*(10) = 0.867, *p* < 0.001, *r*(10) = 0.910, *p* < 0.001, and *r*(10) = 0.981, *p* < 0.001, respectively. The same interviews were also coded for adherence to the interview protocol. Here interviewer performance was rated by two independent coders, naive to the experimental hypothesis, using a scale scoring sheet for each of the aforementioned interview phases, ranging from 1 to 3 (e.g., 1 = fully implemented the greet phase, 2 = partially implemented the greet phase, 3 = did not implement the greet phase). Analysis revealed a substantial level of agreement between raters, Kappa = 0.91, *p* = 0.002. Interviewer adherence across the phases of the aforementioned randomly selected interviews revealed no significant main effects as function of interviewer for adherence to phase 1 (*M*_Interviewer A_ = 1.20; *M*_Interviewer B_ = 1.00), Phase 2 (*M*_Interviewer A_ = 1.20; *M*_Interviewer B_ = 1.20), Phase 3, (*M*_Interviewer A_ = 1.00; *M*_Interviewer B_ = 1.00), Phase 4, (*M*_Interviewer A_ = 1.20; *M*_Interviewer B_ = 1.20), and Phase 5 (*M*_Interviewer A_ = 1.40; *M*_Interviewer B_ = 1.20), all *F*s < 2.667, all *p*s > 0.178.

## Results

### Overall Performance

**FIGURE 3:**
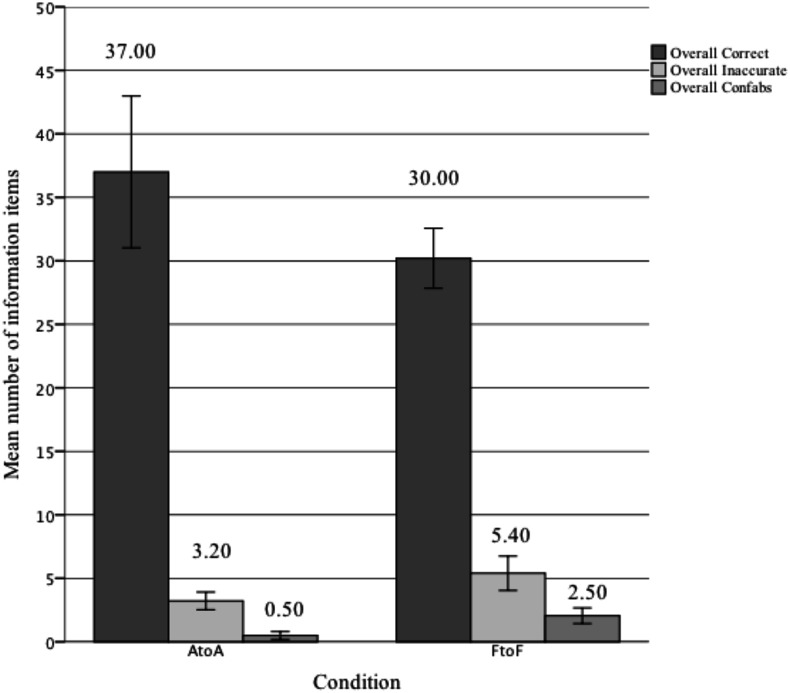
Mean overall episodic performance (with 95% confidence error bars) across retrieval contexts (AtoA and FtoF) for the amount of correct, erroneous, and confabulated information recalled (*N* = 38).

To investigate the overall effect of context (AtoA vs. FtoF) on episodic performance a MANOVA was initially performed on the combination of overall correct, errors and confabulated items recalled (see **Figure 3** for means). This revealed a significant multivariate effect of context on the combination variable, *F*(3,34) = 8.855, *p* < 0.001, $\eta_{p}^{2}$ = 0.44, Pillai’s Trace = 0.439. Overall, participants in the AtoA recalled more correct items, *F*(1,36) = 438.063, *p* = 0.026, $\eta_{p}^{2}$ = 0.13, made fewer errors, *F*(1,36) = 44.932, *p* = 0.006, $\eta_{p}^{2}$ = 0.19, and confabulated less *F*(1,36) = 22.761, *p* < 0.001, $\eta_{p}^{2}$ = 0.36, than those in the FtoF condition.

### Interview Phase Performance

Interviews comprised two distinct retrieval phases: free recall and questioning. Between subject ANOVAs revealed no significant differences across conditions in the first free recall phase for correct items, *F*(1,36) = 3.082, *p* = 0.088, erroneous information, *F*(1,36) = 0.547, *p* = 0.464, or confabulations, *F*(1,36) = 2.173, *p* = 0.149 (see **Table 1**). However, in the questioning phase, participants in the AtoA significantly outperformed those in the FtoF on all measures. They recalled significantly more correct items, *F*(1,36) = 19.352, *p* < .001, $\eta_{p}^{2}$ = 0.35, made fewer errors, *F*(1,36) = 13.956, *p* = 0.001, $\eta_{p}^{2}$ = 0.28, and confabulated less, *F*(1,36) = 24.467, *p* < 0.001, $\eta_{p}^{2}$ = 0.40 (see **Table 1**).

**Table 1:** Mean memory performance (correct, incorrect, confabulations, and percentage accuracy) and 95% CI as a function of condition (AtoA; FtoF) across retrieval phases (*N* = 38).

	A to A	F to F
	Mean (*SD*) [95% CI]	Mean (*SD*) [95% CI]
**Free recall**		
Correct	19.00 (6.60) [15.99; 22.01]	22.00 (4.42) [19.93; 24.07]
Errors	1.44 (1.20) [0.85; 2.04]	1.75 (1.33) [1.13; 2.37]
Confabulations	0.22 (0.43) [0.01; 0.43]	0.50 (0.69) [0.18; 82]
% Accuracy	89.30 (5.57) [86.52; 92.07]	93.12 (6.05) [90.29; 95.96]
**Questioning**		
Correct	18.00 (9.36) [13.35; 22.65]	8.30 (3.00) [6.90; 9.70]
Errors	1.78 (0.88) [1.34; 2.21]	3.65 (1.95) [2.74; 4.56]
Confabulations	0.28 (0.46) [0.05; 0.51]	1.55 (0.99) [1.08; 2.02]
% Accuracy	88.59 (5.57) [85.89; 91.29]	60.10 (13.92) [53.58; 66.62]

### Percentage Accuracy

Overall, AtoA participants were significantly more accurate, (*M*_AtoA_ = 90.30, *SD* = 4.45, CI 95% [88.12; 92.54]), than FtoF participants (*M*_FtoF_ = 73.65, *SD* = 14.98, CI 95% [66.64; 80.66]), *F*(1,36) = 20.634, *p* < 0.001, $\eta_{p}^{2}$ = 0.36. Percentage accuracy between conditions did not differ significantly in the free recall phase *F*(1,36) = 4.070, *p* = 0.051, although percentage accuracy was approaching significance in favor of the FtoF condition. AtoA participants were significantly more accurate in the questioning phase than FtoF participants, *F*(1,36) = 66.153, *p* < 0.001, $\eta_{p}^{2}$ = 0.64. Repeated measures analysis of percentage accuracy as a function of condition revealed no significant difference across retrieval phases for AtoA participants, *p* = 0.674. However, FtoF participants were significantly less accurate in the questioning phase than in the initial free recall phase, *F*(1,19) = 66.157, *p* < 0.001 (see **Table 1**).

### Post-interview Feedback

Analysis of the seven post-interview feedback questions asked of all participants (see **Table 2**) after applying Bonferroni’s correction (resulting in an adjusted alpha of 0.007), four significant differences emerged in reported interview experience (where 1 = Completely disagree; 5 = Completely Agree). AtoA participants reported higher levels of concentration, *F*(1,36) = 30.046, *p* < 0.001, $\eta_{p}^{2}$ = 0.45, CI 95% [4.14; 4.75], found it easier to say when they did not know the answer to a question, *F*(1,36) = 19.747, *p* < 0.001, $\eta_{p}^{2}$ = 0.39, CI 95% [4.04; 4.74], than FtoF participants, CI 95% [2.91; 3.56], CI 95% [2.10; 3.11], and CI 95% [2.92; 3.68], respectively. However, FtoF participants believed they remembered more event information, *F*(1,36) = 14.491, *p* = 0.001, $\eta_{p}^{2}$ = 0.29, CI 95% [3.85; 4.65], and reported being more confident that the information they recalled was correct, *F*(1,36) = 15.405, *p* < 0.001, $\eta_{p}^{2}$ = 0.31, CI 95% [3.63; 4.47], than AtoA participants, CI 95% [2.82; 3.62], and CI 95% [2.74; 3.37], respectively.

**Table 2:** Means (SDs) responses to post-interview feedback as a function of condition (1 = completely disagree; 5 = completely agree).

	Avatar-to-avatar	Face-to-face
	Mean (*SD*)	Mean (*SD*)
The instructions provided by the interviewer/avatar	4.33 (0.67)	4.30 (0.66)
I understood what the interviewer/Avatar was saying to me.	4.28 (0.69)	4.05 (0.61)
The headset/interviewer helped me to concentrate	4.44 (0.62)^∗^	3.25 (0.72)
I found it easy to talk to the interviewer/avatar about what I had	4.11 (2.01)	2.79 (2.10)
It was easy to say ‘ I don’t know’ to the interviewer/avatar	4.39 (0.70)^∗^	3.30 (0.80)
I remembered a lot of information about the	3.22 (0.81)^∗^	4.25 (0.91)
I am confident that the information I remembered	3.06 (0.66)^∗^	4.50 (0.89)

*^∗^p < 0.001*.

Avatar-to-avatar participants were asked an additional three questions (where 1 = Completely disagree; 5 = Completely Agree), as follows: (i) did you experience any negative/adverse effects (e.g., nausea/disorientation) when wearing the Oculus rift headset? (ii) did the headset distract you from remembering what had happened in the video? and (iii) would being able to provide information remotely in an immersive environment be useful for interviewing eyewitnesses across wider contexts. Participants completely disagreed (*M* = 1.32, *SD* = 0.96) that they had experienced any negative effects, completely disagreed (*M* = 1.12, *SD* = 0.76) that the headset was distracting, and agreed (*M* = 4.02, *SD* = 1.06) that this mode of collecting eyewitness testimony would be useful.

## Discussion

We investigated episodic memory performance using the mock witness memory paradigm across two contexts – a traditional face-to-face context, and a computer-mediated context. A limited amount of research has investigated computer-mediated communication for eyewitnesses. For example, typically developing children, and children with Autism Spectrum Disorder recalled more information when interviewed by an avatar than a human (Hsu and Teoh, 2017), and remote interviews via Skype have been found to increase the amount of event relevant information vs. a traditional face-to-face context (Nash et al., 2014; Hamilton et al., 2017). However, as far as we are aware this is the first empirical study of eyewitness cognition in an immersive context where both interviewee and interviewer were represented by avatars.

Overall, AtoA participants interviewed recalled 30% more correct information and made fewer errors than those interviewed face-to-face, which provides support for our hypothesis. Interviews comprised two distinct phases, an initial free recall followed by a witness-compatible questioning phase, and it is memory performance as a function of phase that provides an indication of the locus of the AtoA superiority effect. No differences emerged for any of the performance measures in the free recall phases. However, in the questioning phase, AtoA interviews elicited almost 60% more new correct information items than in the FtF condition, and resulted in significantly fewer errors, and confabulated information, thus improving accuracy. Further, AtoA participants were just as accurate in the questioning phase as in the free recall, conversely participants in the FtoF condition were less accurate across these two phases.

Clearly, the AtoA superiority effect emanated from the questioning phase, a pattern that differs from that typically found in FtoF laboratory eyewitness research (e.g., Stein and Memon, 2006; Dando et al., 2009; Memon et al., 2010; Dando, 2013). Freely recalled witness information is generally more plentiful and the most accurate because no specific questions are asked, and so there is little interviewer involvement (see Fisher and Geiselman, 1992; Milne and Bull, 1999). Hence, witnesses can exert maximum control over their reporting. When witnesses are allowed the freedom to withhold or report information, accuracy is improved (Koriat and Goldsmith, 1996) because they can avoid reporting information they may be unsure about (Goldsmith et al., 2005). In the questioning phase of an interview, however, there is more interviewer involvement. Witnesses are pressed for additional detail about the information reported in the free recall, and the types of questions are numerous and probing (see Milne and Bull, 1999; Griffiths and Milne, 2006; Walsh and Milne, 2008). It is in the questioning phase of an interview that witnesses tend to make more errors of commission (reporting erroneous information) because strategic control is diminished and the cognitive, and social demand (real or imagined) to report more detailed information increases. In laboratory research, where only new information is coded, witnesses typically provide fewer correct information items in this phase, which results in lower accuracy scores. Indeed, this is exactly what we found in the FtoF interviews, but to our surprise, not in the AtoA interviews.

Although the AtoA interviews excluded all external stimuli and reduced the social demands associated with the presence of a human interviewer, this did not impact on performance in this first phase in that participants performed similarly across all memory performance measures. This phase commenced with a free recall account, which supports strategic regulation typically resulting in improved accuracy vs. forced report, or targeted probing, for example (see Koriat and Goldsmith, 1996; Koriat et al., 2000; Dando et al., 2009; Dando, 2013; Paulo et al., 2016). Here, participants in both contexts were able to maintain control of what they reported, and computer-mediated communication neither improved nor diminished their performance in this phase.

It was in the questioning phase, AtoA participants benefitted. It is difficult to unpick whether the reduced social demands afforded by the interviewer appearing as an avatar, or the controlled environment was most beneficial during this phase, or whether these benefits were accumulative. The absence of external stimuli may have supported increased concentration, which can improve eyewitness performance (Vredeveldt et al., 2011; Mastroberardino et al., 2012; Perfect et al., 2012; Vredeveldt and Penrod, 2013). For example, eye-closure has been found to improve the reporting of visual and auditory details by removing the requirement to monitor the retrieval environment (Perfect et al., 2008). Excluding external stimuli changes a dual-task (retrieval and social monitoring) to a single task (retrieval only). Eye-closure can also increase the amount of information provided in cued recall (Mastroberardino et al., 2012). Similarly, the presence of others can affect cognition *per se* (Fiske and Taylor, 2013), and social influence can interfere with witness memory (Steblay, 1997; Wagstaff et al., 2008) albeit social influence is not well understood for episodic performance.

Our pattern of results, whereby memory performance across contexts in the first free recall account did not differ, but was significantly improved in the second more detailed recall phase, indicates that the absence of another *real* person may have been the most important factor for improved performance. Our results support the findings of others that physical co-presence may not be a necessary component of an effective witness interview (see Nash et al., 2014) and that the positive effects of remote interviewing may be as a result of social distance (Doherty-Sneddon and McAuley, 2000). Here, participants were able to choose to be interviewed by either a male or female avatar, but they were dressed in similarly colored and styled clothes, and had the same skin and hair color, etc. (all participants chose the male avatar). We restricted avatar choice, only allowing participants to choose either a male or female avatar to maintain experimental control because research has reported altered behaviors when avatars more closely resemble participants (e.g., Fox et al., 2012; Gonzalez-Franco et al., 2016), for example, which has implications for eyewitness cognition. Varying the appearance of avatar interviewers may further ameliorate performance, particularly for vulnerable witness groups (e.g., children) and those with neurodevelopmental conditions that make social interactions extremely difficult, and which are known to impact upon episodic recall, such as Autism Spectrum Condition.

The avatar may have reduced the social task demands, releasing additional cognitive resources for the task of responding to cued requests. This notion is supported by the post-interview feedback, which reveals that participants found it far easier to say ‘I don’t know’ to the avatar than participants in the FtoF condition who communicated directly with the interviewer. This indicates the social demands experienced by witnesses (see Fisher et al., 2011) were ameliorated by context and the physical absence of the questioner, which may have resulted in improved speaker–listener coordination (Adrianson and Hjelmquist, 1993). Participants did not experience full embodiment in terms of avatar motions, only head movements were tracked, although head movements are viewed as sufficient to exhibit supportive and encouraging interviewer behaviors, which in turn can improve witness remembering (see Fisher and Schreiber, 2017).

Our findings are promising, but there are a number of limitations that must be borne in mind. First, while we have examined the quality and quantity of information recalled, we have not analyzed the *type* of information recalled, nor its forensic utility. It may be that the AtoA and FtoF contexts elicit different types of information. Future work should investigate the type of information recalled across contexts. Second, because the stimulus event depicted a less serious crime and we sought to mirror real life, we had a 48 h delay between encoding and retrieval. Understanding the effect of retrieval context as a function of different delay periods is another avenue for future research. Further, our participant group was drawn from the general population, but they were young adults. Other age groups may perform differently. Developmental variations in episodic memory performance, and the demands of managing the technical and perceptual aspects of recounting an event in a virtual environment may impact on performance for some (e.g., older adults). Finally, we controlled for interviewer variability by keeping the interviewer constant in each of the two conditions. Our two interviewers used a strict protocol, which they adhered to, and they were similarly trained. However, interviewer variability is known to impact on the outcomes of interviews with witnesses and victims, particularly in face-to-face contexts where individual social and verbal behaviors are often unconscious (see Memon et al., 1997; Brown, 2003; Milne and Bull, 2006), and so we cannot rule out that unseen/unknown extraneous interviewer behaviors may have affected our findings.

One commonly accepted principle of a successful interview is the development of rapport. Our interviewers followed a protocol to allow the interviews to be experimentally controlled, which did not include a rapport building phase. Rapport building in VEs is an exciting avenue for future research particularly given the potential for virtual characters to establish rapport through simple contingent non-verbal behaviors (e.g., Bailenson and Yee, 2005; Gratch et al., 2007). Episodic retrieval is facilitated when the context of the crime is recreated at time of recall (Tulving and Thomson, 1973; Fisher, 2010). Currently, best practice guidance in many countries suggests that witnesses should mentally reinstate the context present at encoding prior to recounting an experienced event (see Ministry of Justice (MOJ), 2011; Fisher and Geiselman, 2017). The mental reinstatement of context technique is not appropriate for some witnesses, (see Dando, 2013; Mattison et al., 2015, 2016) who then often underperform. Given the context dependent nature of episodic memory, manipulating VEs to mirror the environmental context to the time of encoding, for example, might improve episodic performance recall still further.

Finally, despite their superior performance, AtoA participants reported feeling less confident about the quality and quantity of their recall performance than participants in the FtF condition. Confidence is not necessarily a predictor of performance accuracy (Deffenbacher, 1980). Confidence can be higher in inaccurate witnesses than accurate witnesses (Wells et al., 1981, 2002), and vice versa (Odinot et al., 2013), and people can confidently recall incorrect information (Sporer, 1992). Overconfidence is an established bias whereby subjective confidence is greater than the objective accuracy, resulting from the need to justify one’s performance (Koriat et al., 1980; Trivers, 1991; Koriat, 2012), or simply because people tend to overestimate their performance (Campbell et al., 2004).

Eyewitness confidence is tractable, and can be affected by context (e.g., Leippe et al., 2009). Future research should consider the social *affect* resulting from the absence of a human interviewer, and whether avatars can reduce over-confidence in eyewitness testimony by controlled social feedback, and if so whether the direction of the relationship can predict memory performance. Manipulating avatar type, may also moderate performance. For example, avatars rated as more attractive to the receiver have been found to moderate social presence and trust (e.g., Jin and Bolebruch, 2009; Chae et al., 2016).

Despite the limitations, this project provides impetus for further research investigating eyewitness cognition in more contemporary retrieval contexts. While the avatar-to-avatar communication element clearly supported performance in a manner that is not as yet not fully understood, this should not detract from our findings. Witness memory is notoriously fragile (Loftus, 2017), but extremely important. Researching eyewitness witness cognition across retrieval contexts may result in highlighting additional effective ‘methods’ for collecting witness information for the criminal justice system.

## Ethics Statement

This study was carried out in accordance with the recommendations of the University of Westminster and British Psychological Society Ethical guidelines. All participants took part in this research on an entirely voluntary basis, and all participants provided written informed consent in accordance with the Declaration of Helsinki. The protocol was approved by the University of Westminster Psychology Ethics Committee.

## Author Contributions

DT and CD designed the study, collected the data, analyzed the resultant data, wrote the Materials and Methods, and Results sections. CD wrote the Introduction section. DT wrote the Discussion section.

## Conflict of Interest Statement

The authors declare that the research was conducted in the absence of any commercial or financial relationships that could be construed as a potential conflict of interest.

## Footnotes

1 From here on we use the term ‘witness’ to refer to witnesses and victims of crime.
